# Rice Novel Semidwarfing Gene *d60* Can Be as Effective as Green Revolution Gene *sd1*

**DOI:** 10.3390/plants8110464

**Published:** 2019-10-30

**Authors:** Motonori Tomita, Keiichiro Ishimoto

**Affiliations:** 1 Research Institute of Green Science and Technology, Shizuoka University, 836 Ohya, Suruga-ku, Shizuoka City, Shizuoka 422-8529, Japan; 2 Faculty of Agriculture, Tottori University, 4-101 Koyama Minami, Tottori 680-8550, Japan

**Keywords:** rice, semidawarf gene, *d60*, *sd1*, yield component, phenotyping, growth

## Abstract

Gene effects on the yield performance were compared among promising semidwarf genes, namely, novel gene *d60*, representative gene *sd1* with different two source IR8 and Jukkoku, and double dwarf combinations of *d60* with each *sd1* allele, in a Koshihikari background. Compared with the culm length of variety Koshihikari (mean, 88.8 cm), that of the semidwarf or double dwarf lines carrying Jukkoku_*sd1*, IR8_*sd1*, *d60*, Jukkoku_*sd1* plus *d60*, or IR8_*sd1* plus *d60* was shortened to 71.8 cm, 68.5 cm, 65.7 cm, 48.6 cm, and 50.3 cm, respectively. Compared with the yield of Koshihikari (mean, 665.3 g/m^2^), that of the line carrying Jukkoku_*sd1* allele showed the highest value (772.6 g/m^2^, 16.1% higher than Koshihikari), while that of IR8_*sd1*, *d60* and IR8_*sd1* plus *d60*, was slightly decreased by 7.1%, 5.5%, and 9.7% respectively. The line carrying Jukkoku_*sd1* also showed the highest value in number of panicles and florets/panicle, 16.2% and 11.1% higher than in Koshihikari, respectively, and these effects were responsible for the increases in yield. The 1000-grain weight was equivalent among all genetic lines. Except for the semidwarf line carrying Jukkoku_*sd1*, semidwarf line carrying *d60* was equivalent to line carrying IR8_*sd1*in the yield of unpolished rice, and yield components such as panicle length, panicle number, floret number /panicle. Therefore, the semidwarfing gene *d60* is one of the best possible choices in practical breeding.

## 1. Introduction

Semidwarfing prevents plants from lodging at their full-ripe stage, making them lodging-resistant to wind and rain, enhances their adaptability for heavy manuring and markedly improved the global productivity of rice and wheat between 1960–1990 (up to double yields of rice and quadruple yields of wheat) [1,2]. Semidwarf rice contributes stable production in the monsoonal regions of Asia, where typhoons frequently occur during the yielding season and also brings benefits such as erect leaf angle, reduced photoinhibition, and possibility to plant at higher densities to japonica varieties grown in California and also in South America [3]. However, gene source of semidwarfness is limited. The International Rice Research Institute (IRRI) developed a semidwarf rice variety IR8 in 1966 by using Taiwanese native semidwarf variety Dee-geo-woo-gen (DGWG). IR8 called as Miracle Rice, has been improved with lodging resistance and light-reception attitude, and it brought the Green Revolution in tropical Asia [2]. In Japan, semidwarf cultivars in the Kyushu region were developed in the 1960s using the native semidwarf variety Jukkoku [4]. In the Tohoku region, semidwarf cultivars were developed in the 1970s using the semidwarf mutant Reimei induced by Fujiminori-gamma-ray irradiated [5]. In the United States, Calrose 76 was developed in 1976 by Calrose-gamma-ray irradiated [6,7].

Genetic study has also been devoted on the genes responsible for semidwarfism in rice. First, a recessive semidwarf gene *d47* was identified in DGWG, the parental line of IR8 [8,9]. Next, the semidwarf gene *sd1* in Calrose 76 was shown to be allelic to *d47* [10,11]. Finally, semidwarf genes in Taichung Native 1 descend from DGWG, Shiranui from Jukkoku, and *d49* in the mutant cultivar Reimei were attributed to the same allele by allelism examination [12,13,14]. Therefore, only a single semidwarf gene, *sd1*, has been commonly used across the world. A little genetic source of current semidwarf rice cultivars have a risk for environmental change. Thus, it is an emerging subject to find a novel semidawrf gene to replace *sd1* and to utilize it to diversify genetic variations of semidwarf rice worldwide.

A novel semidwarf gene, *d60*, which was found in the mutant Hokuriku 100 induced by irradiation of 20 kR of gamma-ray to Koshihikari, is thus of particular importance [15]. While *sd1* is on rice chromosome 1 [16,17], *d60* is located on chromosome 2 (Tomita et al., submitted to Genes). *sd1* is a defective allele encoding GA20-oxidase gene in a late step in the GA biosynthesis pathway [18,19,20]. Moreover, unlike *sd1*, *d60* complements the gamete lethal gene, *gal*. Therefore, in the cross between Hokuriku 100 (*d60d60GalGal*) and Koshihikari (*D60D60galgal*), male and female gametes, in which *gal* and *d60* coexistent, become lethal and the pollen and seed fertility in the F_1_ (genotype *D60d60Galgal*) breakdown to 75%. As a result, the F_2_ progeny exhibits a unique genotype ratio of 6 fertile long-culm (4*D60D60*:2*D60d60GalGal*: 2 partially fertile long-culm (*D60d60Galgal* = F_1_ type):1 dwarf(*d60d60GalGal*) [15].

Although there are multiple alleles in *sd1* locus of DGWG, Jukkoku, Reimei, and Calrose 76, the differences in their influences on the yield performance have not been reported. Therefore, investigating the differences in phenotypic traits among the different *sd1* allele-carrying plants, *d60*-carrying plant and their double dwarf plants, will be beneficial for practical selection of *d60* and *sd1*alleles. In this study, semidwarf or double dwarf lines, which were integrated with *sd1* of Jukkoku, *sd1* of IR8, *d60,* or both gene combinations in the genetic background of Koshihikari, were used for investigating the influence of these semidwarf genes on phenotypic traits, especially related to yield performance.

## 2. Results

### 2.1. Effects of Semidwarf and Double Dwarf Genes on Growth

**Figure 1:**
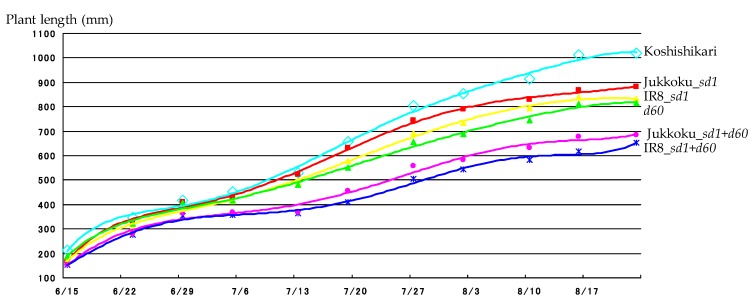
Effect of growth of semidwarf and double dwarf gene lines. Ten plants were randomly selected, and the distance between the ground and the highest standing point (i.e., the full length) was measured every week for approximately three months until the panicle emerged. The full length of *d60*-carrying line was longer than that of *sd1*-carrying lines at the time of transplanting. However, the full length of line carrying Jukkoku_*sd1* and that of line carrying IR8_*sd1* exceeded that of line carrying *d60* on June 23 (43 days after sowing) and on July 13 (64 days after sowing), respectively: full length in lines carrying either Jukkoku_*sd1* or IR8_*sd1* was longer than that in line carrying *d60* at the time of final measurement (August 23, 103 days after sowing).

The trends in full-length growth, depicted by growth curves, were comparable among all lines. (Figure 1). The full length in lines carrying one or two semidwarf genes was already shorter than that of Koshirikari lines at the time of transplanting (June 7, 28 days after sowing), and the differences became prominent around 64–70 days after sowing (July 13 and 19) (Figure 1, Table 1). The full length of *d60*-carrying line was longer than that of *sd1*-carrying lines at the time of transplanting. However, the full length of line carrying Jukkoku_*sd1* and that of line carrying IR8_*sd1* exceeded that of line carrying *d60* on June 23 (43 days after sowing) and on July 13 (64 days after sowing), respectively: full length in lines carrying either Jukkoku_*sd1* or IR8_*sd1* was longer than that in line carrying *d60* at the time of final measurement (August 23, 103 days after sowing). Days to heading ranged from 86.5 days of line carrying IR8_*sd1* to 90.5 days of those carrying *d60*. Such a four-day difference was thought to be little. Therefore, the differences appeared in morphological traits, such as culm length and panicle length, were attributed to genetic reason.

**Figure 2:**
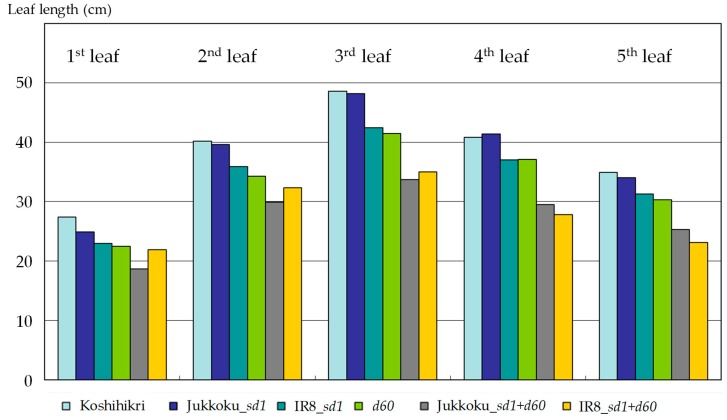
Effect of semidwarf and double dwarf genes to leaf length. Upper five leaves, arising from the main culm, were measured. Except for Jukkoku_*sd1* line. leaves of the semidwarf and double dwarf lines were slightly shorter than that of Koshihikari.

**Figure 3:**
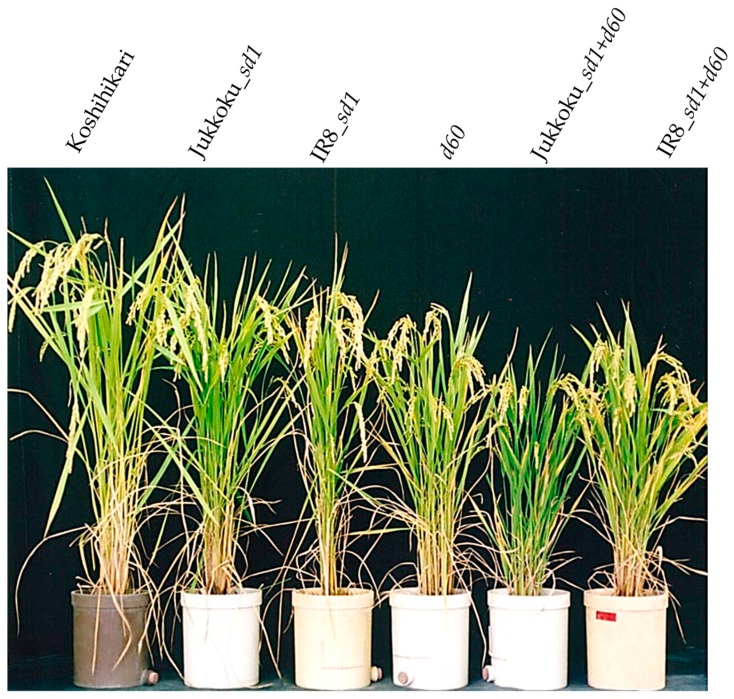
Plant phenotype of semidwarf and double dwarf gene lines. Leaves of the semidwarf and double dwarf lines were straighter (pointing upwards) than in Koshihikari, indicating improved light-reception attitude by the integration of semidwarf gene (or genes).

Integration of a semidwarf gene (or genes) resulted in a reduction in culm length: the mean culm length of Koshihikari was 88.8 cm, while that of lines carrying Jukkoku_*sd1*, IR8_sd1, *d60*, Jukkoku_*sd1* plus *d60*, or IR8_*sd1* plus *d60* was 71.8 cm, 68.5 cm, 65.7 cm, 48.6 cm, or 50.2 cm, respectively. Leaf length was shorter in line carrying Kinuhikari_*sd1* (9–16% reduction compared with Koshihikari) or *d60* (9–18% reduction compared with Koshihikari) than in those carrying Jukkoku_*sd1* (1–9% reduction compared with Koshihikari (Figure 2). Furthermore, leaves of the semidwarf and double dwarf lines were slightly shorter and straighter (pointing upwards) than in Koshihikari (Figure 3), indicating improved light-reception attitude by the integration of semidwarf gene (or genes). Panicle length was slightly longer (by 2.5%) in line carrying Jukkoku_*sd1* and slightly shorter in lines carrying Kinuhikari_*sd1* (by 2.4%) or *d60* (by 3.0%), compared with Koshihikari (Table 2). However, the reduction in panicle length was quite less than that in culm length (22.8% decrease in lines carrying Kinuhikari_*sd1* vs a 26.1% decrease in lines carrying *d60*). Therefore, the negative effects of semidwarf genes *sd1* and *d60* on panicle length were negligible.

### 2.2. Effects of Semidwarf and Double Dwarf Genes on Yield

**Figure 4:**
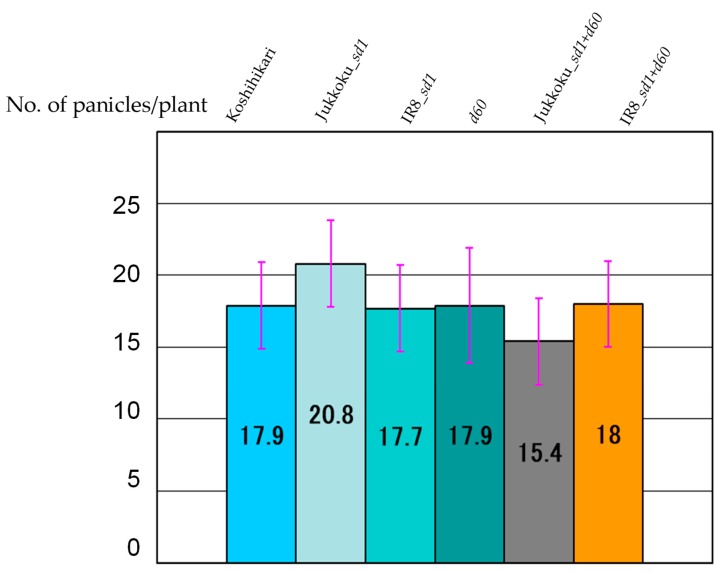
No. of panicles/plant in semidwarf and double dwarf gene lines. The number of panicles/plants was highest at 20.8 in line carrying Jukkoku_*sd1* (+10.2% vs Koshihikari).

**Figure 5:**
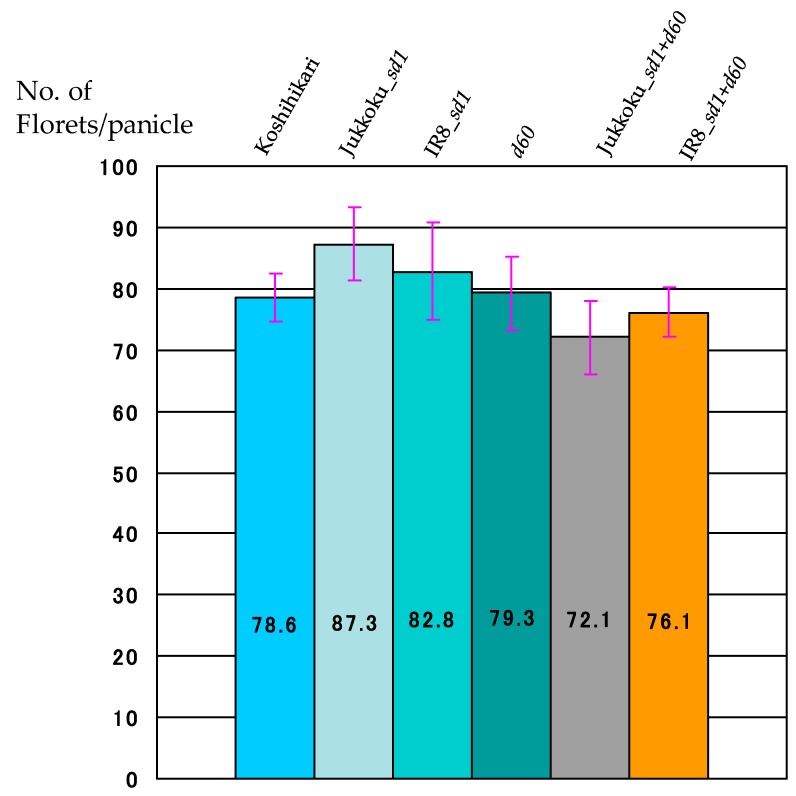
Floret number/panicle of semidwarf and double dwarf gene lines. The number of panicles/plants in line carrying *d60* was comparable to that in line carrying IR8_*sd1*.

**Figure 6:**
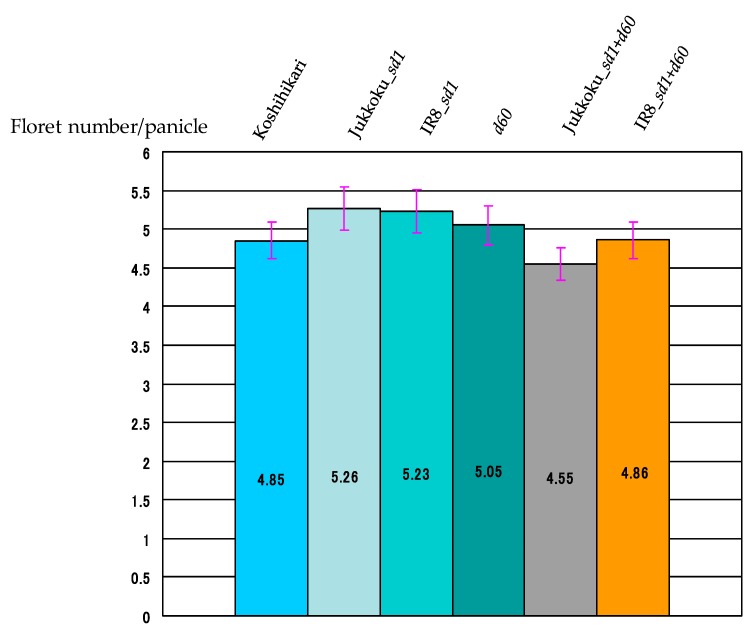
Effect of semidwarf and double dwarf genes to floret density. The floret density was larger in all lines carrying one semidwarf gene than that of Koshihikari.

**Figure 7:**
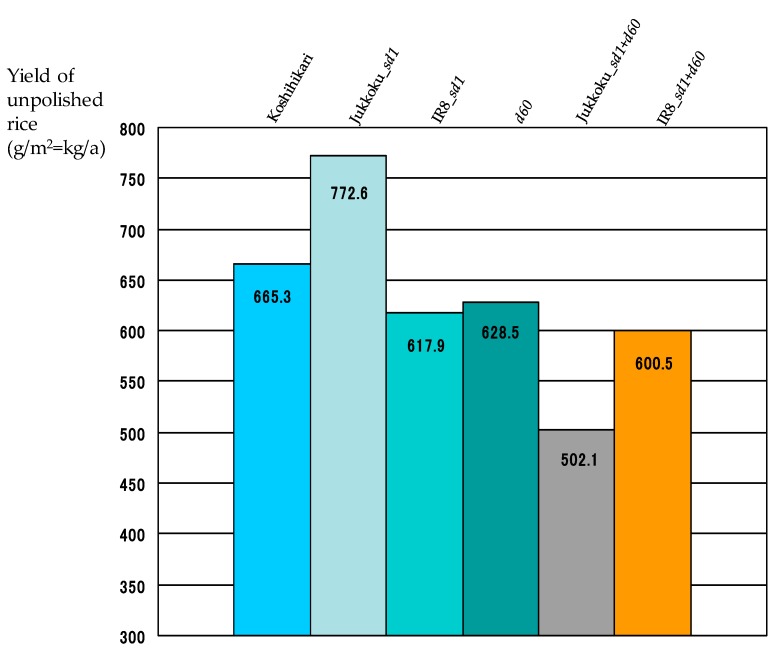
Yield of semidwarf and double dwarf gene lines. The yield of unpolished rice was 665.3 g/m^2^ in Koshihikari, 772.6 g/m^2^ in line carrying Jukkoku_*sd1* (+15.9% vs Koshihikari), 617.9 g/m^2^ in line carrying Kinuhikari_*sd1* (−7.1% vs Koshihikari), and 628.5 g/m^2^ in line carrying *d60* (−5.5% vs Koshihikari).

The yield components of each genotype are summarized in Table 2. The weight of unpolished rice/1000 grains and the proportions of fertile florets differed only slightly between lines. The effect of these genes on the proportion of fertile florets and the weight of unpolished rice/1000 grains were negligible. The number of panicles/plants was 17.9 in Koshihikari: 20.8 in line carrying Jukkoku_*sd1* (+16.2% vs Koshihikari), and 15.4 in Jukkoku DW line (−14.0% vs Koshihikari) (Figure 4, Table 3). In addition, the floret number/panicle was 87.3 in line carrying Jukkoku_*sd1* (+11.1% vs Koshihikari) and 72.1 in Jukkoku DW line (−8.3% vs Koshihikari) (Figure 5, Table 2 and Table 4). The number of panicles was larger in line carrying Jukkoku_*sd1*, while floret density was larger in all semidwarf varieties than in Koshihikari (Figure 6, Table 5). Thus, an increase in both the number of panicles/plant and the floret number/panicle resulted in an increase in the number of panicles/m^2^ and a consequent increase in yield (Figure 7, Table 6).

**Figure 8:**
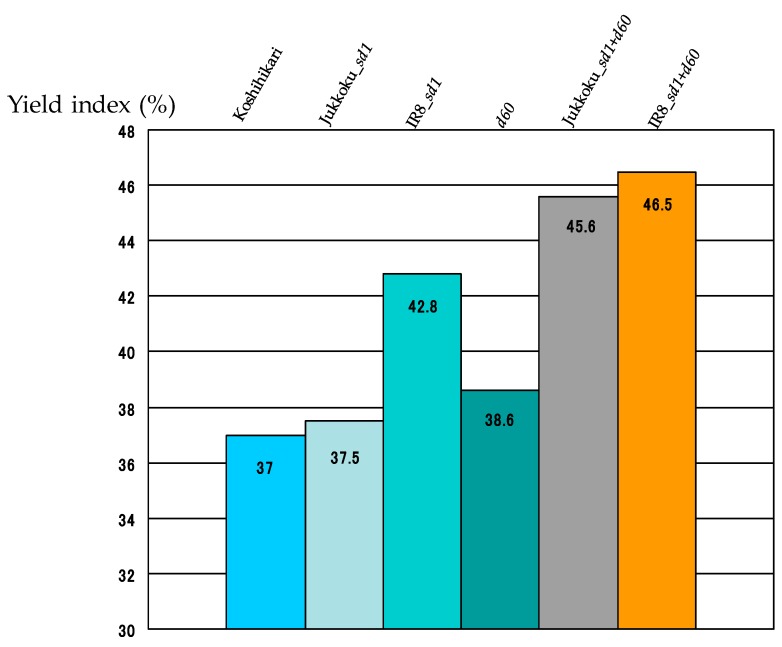
Yield index of semidwarf and double dwarf gene lines. The yield index was higher in line carrying IR8_*sd1* than in those carrying Jukkoku_*sd1.* The yield index for Jukkoku DW and IR8 DW are 45.6% and 46.5%, respectively higher than that of Koshihikari.

The yield of unpolished rice was 665.3 g/m^2^ in Koshihikari, 772.6 g/m^2^ in line carrying Jukkoku_*sd1* (+15.9% vs Koshihikari), 617.9 g/m^2^ in line carrying Kinuhikari_*sd1* (−7.1% vs Koshihikari), and 628.5 g/m^2^ in line carrying *d60* (−5.5% vs Koshihikari) (Figure 7, Table 6). The introduction of Kinuhikari_*sd1* or *d60* into Koshihikari appears to cause a slight reduction in yield. On the other hand, the yield of DW lines was markedly lower than that of Koshihikari: for Jukkoku DW line (−24.5% vs Koshihikari) and 600.5 g/m^2^ for IR8 DW line (−9.7% vs Koshihikari) (Figure 7, Table 6). When using the alternative equation, the yield index was higher in all semidwarf-gene-carrying lines than in Koshihikari (Figure 8). The high yield index and lodging resistance of semidwarf varieties suggest that introduction of *sd1* and *d60* into non-dwarf genomes will be beneficial for increasing crop yield. Moreover, only minor differences in the grain appearance were observed among lines including Koshihikari, indicating that the grain quality of semidwarf lines is equivalent to that of Koshihikari. Taken together, semidwarf genes *sd1* and *d60* are useful in the agricultural industry.

The yield index was higher in line carrying IR8_*sd1* than in those carrying Jukkoku_*sd1* (Figure 8, Table 7). Although line carrying Jukkoku_*sd1* gave a higher yield than those carrying IR8_*sd1*, the higher yield index associated with IR8_*sd1* than Jukkoku_*sd1* suggests that the efficiency of the distribution to sink organs (e.g., seeds) is higher. Thus, the yield favors a gain in dry matter, which may be also higher in plants carrying IR8_*sd1* than in those carrying Jukkoku_*sd1*. Furthermore, the yield index for Jukkoku DW and IR8 DW are high––45.6% and 46.5% higher, respectively than that of Koshihikari (Figure 8, Table 7). In order to increase a markedly low yield in DW lines, the use of conditions that favor a gain in dry matter, such as intensive cultivation with heavy fertilization to increase the number of tillers, may be effective.

## 3. Discussion

As exemplified by IR8, which was the variety behind the Green Revolution, many of the rice varieties cultivated worldwide commonly carry the semidwarf gene *sd1*. Another semidwarf gene *d60* is non-allelic to *sd1* and is of particular interest as a different source of semidwarfism to give genetic diversity among the semidwarf varieties. In this study, semidwarf lines, namely Jukkoku (Jukkoku _*sd1*), *sd1* of Kinuhikari (IR8_*sd1*: Kinuhikari maintains *sd1* of IR8 origin), *d60* or *sd1* plus *d60* into the Koshihikari background, were used to investigate influence of these semidwarf genes on phenotypic traits, in relation to yield.

This study showed that all tested semidwarf lines had shorter culm lengths than Koshihikari, indicating improved lodging resistance. The effect on culm length carrying *d60*(65.7 cm) is slight shorter than in those carrying *sd1* (Jukkoku_*sd1*, 71.8 cm; IR8_*sd1*, 68.5 cm), Among the genetic lines, line carrying Jukkoku_*sd1*showed the highest yield of unpolished rice 772.6 g/m^2^, which is 16.1% higher than in Koshikikari. The Jukkoku_*sd1* line also showed highest value in the number of panicles, the number of florets per panicle than in Koshihikari. Therefore, it was highly possible that the increasing yield of Jukkoku_*sd1* line was ascribed to the increasing numbers of panicles and florets. Although the yield of unpolished rice of *d60* line, 628.5 g/m^2^ was 5.5% lower than that of Koshihikari (665.3 g/m^2^), but this is almost equivalent yield performance of IR8_*sd1* (617.9 g/m^2^). Ogi et al. (1993) [21] and Murai et al. (2004) [22] reported characteristics of isogenic line carrying *sd1* derived from DGWG, the source of IR8 *sd1*. These isogenic lines showed almost same number of panicles as that of original varieties, ‘Norin 29′ and ‘Shiokari’. Therefore, it was concluded that Jukkoku_*sd1* especially has potential increasing panicle numbers compared to IR8_*sd1*. Hence, Jukkoku_*sd1* appears to confer a pleiotropic effect of increasing panicle number very well in the Koshihikari genetic background. The difference of such as effect between *sd1* alleles may be ascribed to that IR8_*sd1* suffered 383 bp deficit in the region exon 1-2 of *GA20-ox* [18], whereas, Jukkoku_*sd1* has only a SNP against the wild type *GA20-ox* [18] and the transcripts existed [23].

This study demonstrated that *d60* confers slightly shorter culm length than IR8_*sd1*, but almost equivalent yield performance with IR8_*sd1* together with effects on yield-related phenotypic traits comparable to IR8_*sd1*, which actually contributed to green revolution [2]. Although many dwarf genes are accompanied with a reduction in panicle length, yield of unpolished rice, and grain thresh ability (which is likely attributed to excessive dwarfing effects), *d60* does not exert such negative effects on yield-related phenotypic traits of rice plant. In conclusion, *d60* is applicable to practical breeding and one of choice for expanding genetic diversity of rice varieties.

## 4. Materials and Methods

### 4.1. Genetic Lines

The following rice semidwarf or double dwarf lines, Koshihikari, Koshihikari carrying Jukkoku_*sd1* [Koshihikari*6//(Kanto 79/Jukkoku F_4_) B_6_F_4_], Koshihikari carrying IR8_*sd1* [Koshihikari/Kinuhikari F_5_)], Koshihikari carrying *d60* [Koshihikari Koshihikari*7//(Koshihikari/Hokuriku 100) B_7_F_3_], Koshihikari carrying *d60* and Jukkoku_*sd1* [Jukkoku_DW, Koshihikari carrying Jukkoku_*sd1*/Koshihikari carrying_*d60* F_7_), and Koshihikari carrying *d60* and IR8_*sd1* (IR8 DW: Koshihikari carrying IR8_*sd1/* Koshihikari carrying_*d60* F_7_) were used in this study. Koshihikari carrying Jukkoku_*sd1* was developed by six times of backcrosses with Koshihiakri as a recurrent parent using the short stemmed *sd1* homozygous fixed F_4_ strain in Kanto No. 79 × Jukkoku (*sd1*) as a non-recurrent parent [24]. Koshihikari carrying *d60* was developed by seven times of backcrosses with Koshihikari as the recurrent parent using the short-stemmed F_2_ plant as a non-recurrent parent [15]. Koshihikari carrying IR8__*sd1* was the short stemmed *sd1* homozygous fixed F_7_ strain derived from Koshihikari × Kinuhikari. Koshihikari carrying *d60* and Jukkoku_*sd1*(Jukkoku DW) was double dwarf *d60sd1* homozygous fixed F_7_ strain derived from Koshihikari carrying Jukkoku_*sd1* × Koshihikari carrying_*d60* [15]. Koshihikari carrying *d60* and IR8_*sd1*(IR8 DW) was double dwarf *d60sd1* homozygous fixed F_7_ strain derived from Koshihikari carrying IR8_*sd1* × Koshihikari carrying_*d60* [15]. Kinuhikari has *sd1* derived from IR8 [25], which suffer 383 bp deficit in the region exon 1-2 from wild type *GA20-ox* [18]. Koshihikari carrying Jukkoku_*sd1*was plant-variety registered via further 7–8th backcrosses and it was designated as Hikarishinseiki [24], whose *sd1* has only a SNP against wild type *GA20-ox* [18,26]. Genomic *sd1*allele and the RNA transcript in Hikarishinseiki are detectable by diagnosis targeting the SNP [23,27].

### 4.2. Cultivation

Rice seeds were taken from stocks kept in a refrigerator. Seeds of each line were immersed in enough water just to cover the seeds. Water was exchanged every day for seven days (May 2 to May 8) during seed soaking and stimulation of germination. Seedlings were grown in nursery boxes (30 × 15 × 3 cm) for approximately 20 days. Seedlings were then individually transplanted into a paddy field (120 m^2^: 6.0 × 20.0 m) of the University Farm on June 8. Two 4-m^2^ plots (2 × 2 m) with transplanting densities 22.2 seedlings/m^2^ (one seedling per 30 × 15 cm, 78 seedlings per field) were prepared for each genetic line (two instances). The paddy field was fertilized by 4.0 kg of basal fertilizer containing nitrogen, phosphorus, and potassium (weight ratio, nitrogen:phosphorus:potassium = 2.6:3.2:2.6) at the rate with 4.3 g/m^2^ nitrogen, 5.3 g/m^2^ phosphorus, and 4.3 g/m^2^ potassium evenly across the field. A herbicide (Joystar L Floable, Kumiai Chemical Industry, Tokyo, Japan) was applied on June 20 to kill weeds grown uncontrollably, and the water level was then kept at a high enough level to cover the weeds for a week.

### 4.3. Growth Analysis

Ten seedlings were randomly selected for each line at the time of transplantation, and the full length was measured individually. After transplantation, ten plants were randomly selected, and the distance between the ground and the highest standing point (i.e., the full length) was measured every week for approximately three months until the panicle emerged. The time when the tip of the panicle first emerged from the flag leaf sheath was recorded as the heading time for all plants.

### 4.4. Plant Phenotyping

After ripening, ten plants typical of each genotype were sampled twice. Sampled plants were air-dried, and were assessed or measured the following traits. Culm length: the length between the ground surface and the panicle base of the main culm was measured at the time of sampling. Leaf length: the lengths of the upper five leaves, arising from the main culm, were individually measured. Length and weight of panicle: the length between the panicle base and the tip of the panicle, and the weight of the panicle, were measured. Total panicle number: the number of panicles were counted by sampled individuals and panicle numbers per 1 m^2^ area (panicles/m^2^) were counted twice in each plot of the paddy field. Total floret number: florets were counted to obtain total floret number. Floret number/panicle: the total floret number (including both sterile and fertile florets) was divided by the total panicle number. Proportion of fertile florets: each floret was assessed to determine its fertility. Floret density (floret number/cm): the number of florets per panicle was divided by the length of the panicle. Presence of awns: florets with an awn were counted when counting the florets. Grain threshability: was manually tested during examination of phenotypic traits. Appearance of grains: the size, color, and presence of an awn were observed for assessment of grain quality. Total weight of winnowed paddy: the total weight of winnowed paddy was weighed after grain selection using the salt solution (salt content of 1.06 g/m^3^). Weight of sieved unpolished rice/1000 grains: obtained by multiplying the total weight of winnowed paddy by 0.84. Weight of plant parts above the ground: the weight of the plant parts above the ground was measured. Yield index: the winnowed paddy weight was divided by the weight of the plant part above the ground to obtain the yield index. The means of traits were statistically compared using the *t*-test.

### 4.5. Yield

Yield of unpolished rice was calculated using the following equation.

- Yield of unpolished rice (g/m^2^) = (number of panicles/m^2^) × (floret number/panicle) × (proportion of fertile florets) × (weight of unpolished rice/grain)

The following alternative equation (see below) was also used to calculate the comparison of yields:

- Yield = (yield index) × (weight of the plant parts above the ground)

## Figures and Tables

**Table 1:** Plant length of semidwarf and double dwarf gene lines.

Days after Sowing	35	43	49	55	64	70	78	82	90	96	103
**Koshihikari**	212.6	345.0	415.4	452.4	529.5	657.0	805.6	852.6	913.1	1011.6	1020.3
**Jukkoku_*sd1***	166.8 *	330.2	408.9	430.7	523.8	631.0 *	743.0 *	791.7 *	828.7 *	865.9 *	880.8 *
**IR8_*sd1***	170.3 *	308.9 *	393.2	414.9 *	488.8 *	578.2 *	693.2 *	734.4 *	794.6 *	837.9 *	831.0 *
***d60***	189.1 *	321.0 *	406.5	415.9 *	480.5 *	550.4 *	656.8 *	689.3 *	744.1 *	810.6 *	814.3 *
**Jukkoku_*sd1*+*d60***	153.1 *	275.4 *	352.8 *	357.8 *	364.1 *	409.2 *	506.4 *	543.4 *	581.9 *	616.6 *	651.7 *
**IR8_*sd1*+*d60***	153.7 *	279.2 *	364.8 *	368.5 *	369.1 *	454.5 *	558.4 *	580.9 *	630.9 *	679.2 *	684.2 *

The full length in lines carrying one or two semidwarf genes was already shorter than that of Koshirikari lines at the time of transplanting 28 days after sowing, and the differences became prominent around 64–70 days after sowing. Finally, the full length of semidwarf and double dwarf lines were significantly shorter than Koshihaikri. Color in the boxes of genetic lines coincide the color of growth curve in Figure 1. *: statistically significant at the 5% level.

**Table 2:** Effect of semidwarf and double dwarf genes to yield components.

	Koshihikari	Jukkoku_*sd1*	IR8_*sd1*	*d60*	Jukkoku _*sd1*+*d60*	IR8_*sd1*+*d60*
Weight of unpolished rice/1000 grains (g)	22.1	20.2	20.4	20.9	21.3	20.5
Panicles /m^2^	397.4	461.8 *	391.8	397.4	341.9 *	399.6
Floret number/panicle	78.6	87.3 *	82.8	79.3	72.1 *	76.2
Seed fertility (%)	96.6	94.9	93.4	95.4	95.6	96.2
Yield of unpolished rice (g/m^2^=kg/a)	665.3	772.6 *	617.9 *	628.5	502.1 *	600.5 *

Compared with the yield of Koshihikari (mean, 665.3 g/m^2^), that of the line carrying Jukkoku_*sd1* was highest value 772.6 g/m^2^ increased by 16.1%, while that of IR8_*sd1*, *d60* and IR8_*sd1* plus *d60*, was slightly decreased by 7.1%, 5.5%, and 9.7%, respectively. The line carrying Jukkoku_*sd1* also showed highest value in number of panicles and florets/panicle, each 16.2% and 11.1% higher than in Koshihikari, which were responsible for the increases in yield. The weight of rice/1000 grains was equivalent among all genetic lines. Except for the semidwarf line carrying Jukkoku_*sd1*, semidwarf line carrying *d60* was equivalent to line carrying IR8_*sd1*in the yield of unpolished rice, and yield components such as panicles/m^2^, floret number /panicle. *: statistically significant at the 5% level.

**Table 3:** Effect of semidwarf and double dwarf genes to No. of panicles/plant.

	Koshihikari	Jukkoku_*sd1*	IR8_*sd1*	*d60*	Jukkoku _*sd1*+*d60*	IR8_*sd1*+*d60*
No. of panicles/plant	17.9	20.8 *	17.7	17.9	15.4 *	18.0
Percent change (%)	-	+16.2	−1.4	±0	−14.0	+0.6

The number of panicles/plants in line carrying *d60* (17.9) was comparable to that in line carrying IR8_*sd1* (17.7). *: statistically significant at the 5% level.

**Table 4:** Effect of semidwarf and double dwarf genes to floret number/panicle.

	Koshihikari	Jukkoku_*sd1*	IR8_*sd1*	*d60*	Jukkoku _*sd1*+*d60*	IR8_*sd1*+*d60*
No. of Florets/panicle	78.6	87.3 *	82.8	79.3	72.1 *	76.1
Percent change (%)	-	+11.1	+5.3	+0.9	−8.3	−3.1

The floret number/panicle was highest at 87.3 in line carrying Jukkoku_*sd1* (+11.1% vs Koshihikari). *: statistically significant at the 5% level.

**Table 5:** Effect of semidwarf and double dwarf genes to panicle.

	Koshihikari	Jukkoku_*sd1*	IR8_*sd1*	*d60*	Jukkoku_*sd1*+*d60*	IR8_*sd1*+*d60*
Floret number/panicle	78.6	87.3 *	82.8	79.3	72.1 *	76.1
Panicle length (cm)	16.2	16.6	15.8	15.7	15.8	15.7
Floret density (/cm)	4.85	5.26 *	5.23 *	5.05	4.55 *	4.86
Percent change of floret density (%)	-	+8.53	+7.95	+4.11	−6.15	+0.32

Panicle length was slightly longer (by 2.5%) in line carrying Jukkoku_*sd1* and slightly shorter in lines carrying IR8_*sd1* (by 2.4%) or *d60* (by 3.0%), compared with Koshihikari. The reduction in panicle length was quite less than that in culm length (22.8% decrease in line carrying IR8_*sd1* vs a 26.1% decrease in line carrying *d60*).

**Table 6:** Effect of semidwarf and double dwarf genes to yield.

	Koshihikari	Jukkoku_*sd1*	IR8_*sd1*	*d60*	Jukkoku _*sd1*+*d60*	IR8_*sd1*+*d60*
Yield of unpolished rice (g/m^2^=kg/a)	665.3	772.6 *	617.9 *	628.5	502.1 *	600.5 *
Percent change (%)	-	+16.1	−7.1	−5.5	−24.5	−9.7

The yield of line carrying *d60* was comparable to that in line carrying IR8_*sd1*. *: statistically significant at the 5% level.

**Table 7:** Effect of semidwarf and double dwarf genes to yield index.

	Koshihikari	Jukkoku_*sd1*	IR8_*sd1*	*d60*	Jukkoku _*sd1*+*d60*	IR8_*sd1*+*d60*
Weight of winnowed paddy (g)	26.9	27.3	26.4	22.7 *	20.3 *	23.9 *
Weight of the plant part above the ground (g)	72.7	72.8	61.5 *	58.9 *	44.6 *	51.4 *
Yield index (%)	37.0	37.5	42.8 *	38.6	45.6 *	46.5 *

The high yield index and lodging resistance of semidwarf varieties suggest that introduction of *sd1* and *d60* into non-dwarf genomes will be beneficial for increasing crop yield. *: statistically significant at the 5% level.
